# Orbit Image Analysis: An open-source whole slide image analysis tool

**DOI:** 10.1371/journal.pcbi.1007313

**Published:** 2020-02-05

**Authors:** Manuel Stritt, Anna K. Stalder, Enrico Vezzali

**Affiliations:** Scientific Computing Biology, Idorsia Pharmaceuticals Ltd, Allschwil, Switzerland; Broad Institute of MIT and Harvard, UNITED STATES

## Abstract

We describe Orbit Image Analysis, an open-source whole slide image analysis tool. The tool consists of a generic tile-processing engine which allows the execution of various image analysis algorithms provided by either Orbit itself or from other open-source platforms using a tile-based map-reduce execution framework. Orbit Image Analysis is capable of sophisticated whole slide imaging analyses due to several key features. First, Orbit has machine-learning capabilities. This deep learning segmentation can be integrated with complex object detection for analysis of intricate tissues. In addition, Orbit can run locally as standalone or connect to the open-source image server OMERO. Another important characteristic is its scale-out functionality, using the Apache Spark framework for distributed computing. In this paper, we describe the use of Orbit in three different real-world applications: quantification of idiopathic lung fibrosis, nerve fibre density quantification, and glomeruli detection in the kidney.

## Author summary

Whole slide images (WSI) are digital scans of samples, e.g. tissue sections. It is very convenient to view samples in this digital form, and with the increasing computation power it can also be used for quantification. These images are often too large to be analysed with standard tools. To overcome this issue, we created on open-source tool called Orbit Image Analysis which divides the images into smaller parts and allows the analysis of it with either embedded algorithms or the integration of existing tools. It also provides mechanisms to process huge amounts of images in distributed computing environments, such as clusters or cloud infrastructures. In this paper we describe the Orbit system and demonstrate its application based on three real-word use-cases.

This is a *PLOS Computational Biology* Software paper.

## Introduction

The use of digital pathology (DP) as a companion diagnostic is growing rapidly, with several reports of pathology departments transitioning towards signing out cases using either partial or (in some cases) completely digital workflows [1,2,3,4]. This trend, along with the availability of commercial whole slide image (WSI) scanners, has helped to drive the adoption of DP as both a research and clinical tool. However, the purpose of acquiring WSI digital data is not just to have a digital, computer-aided display of pathology specimens, but to make it possible to apply advanced computer vision and machine learning tools. Ideally, this will accelerate the processing of slides and cases in research, drug development, clinical trials and patient diagnosis—across all the domains where pathology makes a critical contribution to our understanding of the effects of experimental perturbation or disease.

An extensive set of algorithms with conventional and deep learning tools is available today either in commercial or open-source form. Furthermore, the community that developed these tools has matured, so many tools can now be applied to real-world use cases [5,6]. This maturation has made it possible to build user-facing software platforms that deliver these advanced algorithms in the form of convenient, user-directed programs from either commercial or open-source developers. The first generation of these algorithms was founded on intensity-based pixel classification to quantify images. While this worked well for simple analysis tasks, for slightly more complex tasks, they failed completely. In lung fibrosis quantification, for example, normal structural collagen has the same staining colour as newly-deposited fibrotic collagen. These earlier algorithms were not able to detect such differences, whereas for pathologists these tasks were relatively straightforward. The reason for this discrepancy is that humans can take the tissue context into consideration, e.g. to understand that a cell lies within a particular region of a tissue and thus is different from another cell, even if both have the same colour. Simple intensity-based pixel analysis cannot deliver an acceptable result in this case. Instead, a more holistic approach is required that combines intensity and colour recognition with more sophisticated features that can quantify the context of any object, and include this context in the classification in order to successfully analyse samples of lung fibrosis or other challenging tissues. Many feature-based approaches at the object- or cell-level have been published to describe specific-use scenarios [7,8,9]. The emergence of deep learning methods has facilitated the quantification of even more complex scenarios [10,11,12,13,14].

To harmonize these approaches and create a generic and modular framework for sophisticated WSI quantification, we developed Orbit Image Analysis. This framework combines machine learning to understand the context within extensive WSI, and leverages this knowledge to analyze structures at different magnification levels. In addition, the Orbit framework allows the integration of arbitrary analysis algorithms including deep learning. Orbit is an open-source software, with the binary executables and source code freely available (https://www.orbit.bio/; https://github.com/mstritt/), licensed under GPLv3. A community forum for discussions and requests is available at https://forum.image.sc/tags/orbit. In this paper, we describe the development of the Orbit Image Analysis tool and its application in three real-world scenarios.

## Design and implementation

Orbit was developed in Java in order to be platform-independent and therefore can run under Linux, Mac, and Windows. Its user interface is implemented in Swing, but it can also run headless from the command-line. This is a key feature that enables server-side (batch) processing, e.g. in the cloud or on an Apache Spark cluster.

Orbit’s context-based structure classification is based on the so-called structure-size, a surrounding area for each pixel, which is used to compute several features across multiple image resolutions. These features describe the structure of the underlying tissue or other biological sample and are used as an input for a linear Support Vector Machine (SVM) to discriminate regions within the image. In contrast to deep learning methods where a huge training set is needed, this approach allows users to specify just a few training regions on-the-fly and create a model within minutes. This method can be compared to the mechanism of ilastik [15], a pixel classifier tool which also provides methods for object segmentation and classification, or the trainable segmentation provided by Fiji [16]. In contrast to these methods, however, Orbit has been developed to cope with very large images, such as WSI. To do so, Orbit processes the images in a tile-based manner and can handle several resolutions.

In addition to this basic functionality, sophisticated image analysis algorithms, such as segmentation and deep learning classification exist in the open-source world. Nevertheless, these algorithms usually work on small images that fit within the memory, but not on very large images. This is an important limitation because WSI are typically quite large, e.g., 150,000 x 60,000 pixels and recent advances in staining and data acquisition can produce multiplexed images of these dimensions across 40 different channels or more [17]. Orbit’s generic map-reduce based tile-processing approach enables the application of these algorithms towards large WSI.

The combination of the context-based structure classification together with the generic tile-processing engine which enables the integration of further algorithms has yielded a comprehensive, scriptable and scalable framework for objective WSI quantification.

Another powerful WSI tool is QuPath [18], which focuses on object segmentation in combination with an object-based data structure which allows further analysis on the segmented objects. Its sophisticated data analysis and visualization methods have been mainly targeted towards bright-field images. QuPath provides a practical tool chain starting from Tissue Micro Array (TMA) processing up to visualization of the results. SuRVoS [19] is an image analysis tool which can also handle very large images, but focuses on volumetric analysis. Cytomine [20] is a web-based tool which provides sophisticated functionality to enable collaborative work on images, e.g. to create and share annotations. In contrast to these tools, Orbit’s main focus is to enable the use of existing image analysis tools for performing WSI. To this end, Orbit provides a generic tile-processing engine such that algorithms can be easily integrated within its system. In addition, it comes with sophisticated built-in tools ready to use within a user-friendly interface. The presence of a batch mode enables the analysis of hundreds of images in parallel on a scale-out infrastructure, e.g. an Apache Spark cluster which allows image processing on an on-premise cluster or using cloud services, such as Amazon’s EMR. The Orbit system is designed for two user groups: image analysis specialists and other end-users, e.g. pathologists. Image analysis specialists can script the system, use the Application Program Interface (API) in scripts, or write new modules for it. Other end-users can work within the user interface which provides out-of-the-box functionality for many use-case scenarios. We started building Orbit Image Analysis in 2009 and have successively incorporated more functionality by constantly applying it directly to real world problems. Now, Orbit is able to solve most image analysis tasks related to the drug discovery domain.

We present the application of this tool towards solving three real-world problems. Each of these applications makes use of the map-reduce [21] based tile-processing approach of Orbit. First, the mapper step applies image analysis algorithms on tiles (which can be performed in parallel), and the reducer step combines the tile results. Orbit provides functionality for pixel classification, object segmentation and classification, and deep learning methods for complex heterogeneous object detection. These algorithms can be applied towards different resolutions of the image which allows combined processing of different image context levels. This is comparable to the way a human pathologist works: in general, pathologists begin with a low magnification objective to get an overview and analyse the tissue context, and only then switch to a high magnification objective to analyse part of the tissue in detail while keeping the broader context in mind.

Orbit Image Analysis is a versatile tile-processing engine for whole slide imaging. It is a modular system which can access image and metadata through several image providers, apply image analysis algorithms in a map-reduce manner, and optionally use a scale-out infrastructure like Apache Spark to execute intensive tasks in a timely fashion, by using a distributed compute environment. Out-of-the-box, it can connect to the open-source image server OMERO, or run in stand-alone mode to access local files from the hard disk. Orbit has many machine-learning based algorithms built-in such as pixel classification, object segmentation and object classification. A sophisticated deep learning segmentation method based on a DeepLab v2 (ResNet-101) network architecture [22] pre-trained on the Common Objects in Context (COCO) dataset [23] allows the detection of complex heterogeneous objects. Additional algorithms can be implemented easily and integrated as modules. Orbit comes with a script editor which allows the execution of Orbit scripts directly inside the user interface. This open-source program and some models like the glomeruli detection model are licenced under the GPLv3 and can be downloaded at https://www.orbit.bio.

### Architecture

**Fig 1:**
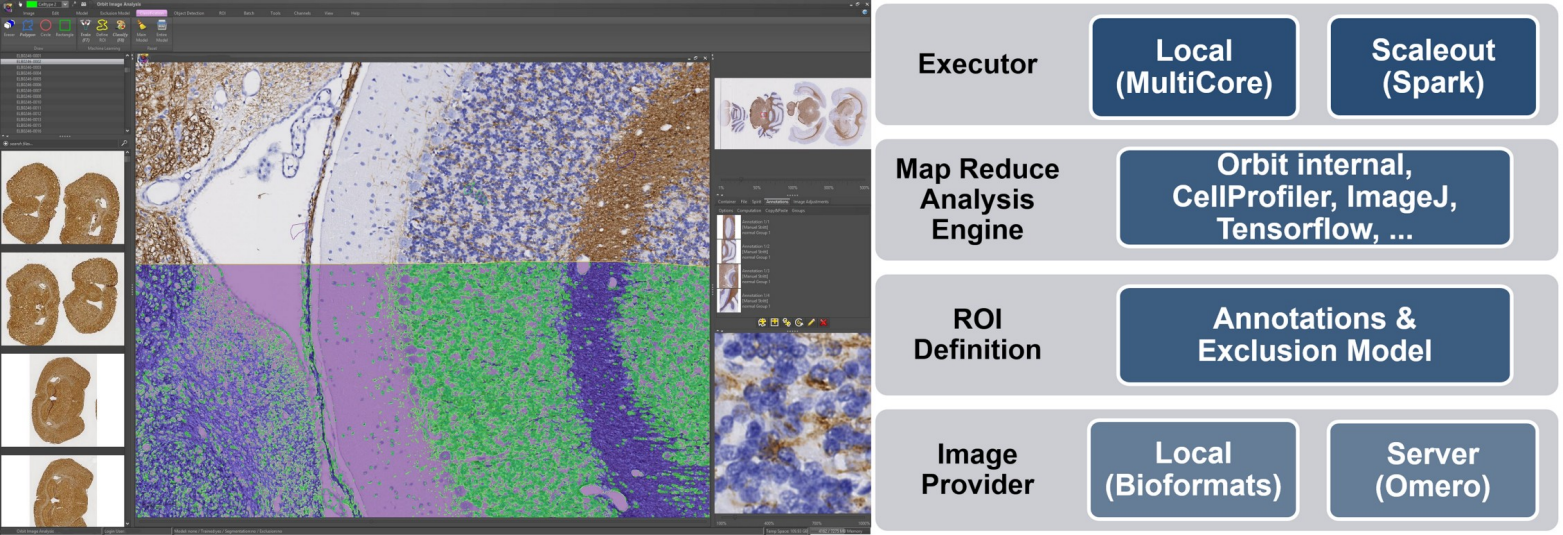
Overview of Orbit. Left: The Orbit user interface: Image browser on the left, tasks are on top grouped in tabs; properties and working results are on the right. Image viewer in the centre. Right: The architecture of Orbit Image Analysis.

Orbit Image Analysis is a modular system which allows the exchange of several components. Fig 1 shows an overview of the architecture.

#### Image provider

Image providers supply tile-based image data, provide search functionality, and read and store metadata like annotations and models. Out-of-the-box, Orbit comes with a local image provider which reads images from the disk in standalone mode. The *BioFormats* library from the Open Microscopy Environment (https://www.openmicroscopy.org/) is used to read many proprietary WSI file formats. A unique ID based on the md5 hash of the image data is assigned to each image. Metadata is stored in a SQLite database on the local disk. In addition, the image provider OMERO can be used to connect to the open-source image server OMERO [24]. This image provider makes use of the OMERO-API to read tile-based image data and to store metadata. The authentication mechanisms and access rights of OMERO are used in this context. This allows a collaborative workflow in a distributed environment, e.g. biologists can create annotations, and image analysis experts can run analysis algorithms within these annotations. Other image providers can be easily integrated by implementing an *IImageProvider* interface so that other imaging infrastructures can be used as a data source.

#### Region of interest definition

For WSI, definition of the region of interest (ROI) is very important. This defines e.g. where the tissue on the slide is located, or more specifically, which parts of a tissue should be analysed. For this, Orbit provides two ROI definition modules. First, manual annotations can be drawn using the annotation tool panel. Several tools including a ‘magnetic lasso’ can be used to define a combination of ROIs, exclusion regions and explicit inclusion regions (areas which will be included even if excluded by other rules like exclusion annotations). Second, a so-called exclusion model can be trained. This is a pixel-based machine-learning model trained on a lower resolution of the image to define inclusion and exclusion areas. This powerful feature greatly speeds up tedious manual annotation tasks. Such an automated approach can be combined with the manual annotations, which have priority.

#### Map-reduce paradigm

Image processing within Orbit is performed in map-reduce manner. The map step is performed at the tile level; the reduce step aggregates all results from the map step. This allows arbitrary algorithms, e.g. ImageJ [25], Fiji [16], or Cell Profiler [26], to run on small in-memory image tiles, even if the algorithm is not designed for WSI. A good example is cell count: the map step counts cells in each tile (e.g. 512x512 pixel), the reduce step is simply a sum function. The advantage of this process is that the map steps can be executed independently of one another, enabling it to be executed in a multi-threaded manner on a local computer or distributed, e.g. using an Apache Spark environment. Orbit comes with built-in algorithms like an advanced structure-based pixel-classification, object segmentation and object classification. In addition, other algorithms or tools like Cell Profiler, ImageJ Macros, or Deep Learning segmentation using TensorFlow [27] can be used in the map step.

This generic map-reduce framework was developed in order to allow the implementation of map and reduce steps, followed by the execution of these steps using different executors. Orbit comes with a local executor which performs map-reduce tasks multithreaded on the local computer. In addition, an executor for Apache Spark can be used to execute tasks on a cluster, if available. This scale-out functionality allows the use of on-premise clusters with a Spark installation, or the use of cloud-based services, such as Amazon’s EMR. Additional executors can be developed easily by implementing the *IMapReduceExecutor* interface.

### User interface

The user interface facilitates easy model and annotation creation and can be used as an image viewer for WSI. On the left panel, datasets and images can be searched and selected. The image provider defines the hierarchical structure, e.g. in OMERO images are arranged by groups, projects and datasets. A search field allows a quick search by filename.

The top tabs show tasks like browsing and saving images and models, image analysis tasks (violet tabs), batch processing, plugin extensions (tools) and a versatile Groovy script editor. Each task is organized in several ribbon buttons which usually should be applied in the order from left to right. The ribbons with bold font are mandatory steps, the others optional. A mouse-over help tooltip is displayed for each button which should limit the need for additional documentation. A handbook and tutorials are available under https://www.orbit.bio/help/.

The right panel shows working elements, such as annotations and models, and image-specific tools such as image adjustments and metadata. For instance, the annotation tab provides the possibility to draw ROIs, which may then be optionally stored in the database.

In the central pane, images and mark-up are rendered. The Orbit render engine supports standard RGB (bright-field) and multi-channel (fluorescence) images. In the latter case each channel is assigned a hue value, which can be modified by the user in the *Adjustments* panel. The render engine is multi-threaded, so that several tiles of the image are rendered off-screen in parallel and then displayed when the render process has finished. Orbit makes use of image pyramids for fast rendering of large images at low zoom levels. Several overlays like a classification mask, segmented objects or manual annotations can also be rendered on top of the image.

#### Script editor

**Fig 2:**
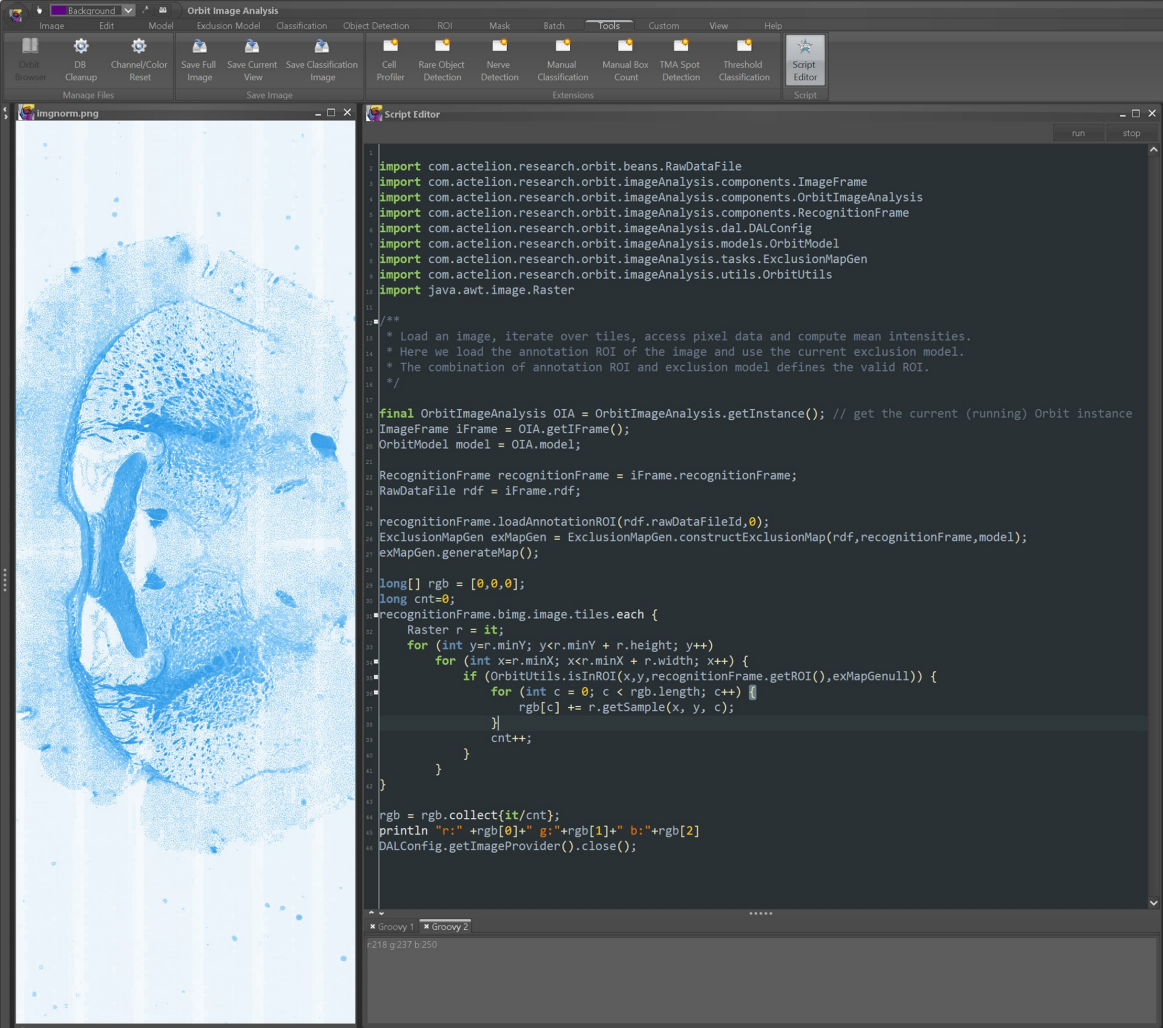
Integrated script editor. The integrated script editor allows the execution of Groovy scripts which can access the Orbit API. Results can be visualized as a mark-up on open image frames.

An integrated Groovy script editor enables the execution of Groovy Script directly in Orbit (Fig 2). The advantage of the integrated editor is that the code execution runs within the same Java Virtual Machine (JVM) and thus can access all active objects and has the full Orbit plus dependencies class-path available. The main controller class *OrbitImageAnalysis* is a singleton and can be used to easily access all open images and models. It also can read and process all image data and even write back objects, e.g. polygons or an overlay map.

Scripting allows the automated execution of manually performed steps in the user interface, and in addition enables the execution of complex analysis steps which are not integrated within the user interface. Orbit provides sophisticated helper methods in the *OrbitHelper* class to handle ROIs, classify pixels, segment objects or classify objects. Image data can be requested to execute arbitrary algorithms on tile data, e.g. to run an ImageJ macro on it, which enables the full power of such libraries to be harnessed at the tile level.

The benefit of the script editor is that the workload of image analysis specialists and end-users can be split: the more programming-oriented image analysis specialists can write parameterized scripts so that the end-users can adapt some parameters (e.g. a threshold), and without requiring deep knowledge of scripting, run the script, validate the results, and adjust the parameters if required. The validation can be done visually if the script writes segmentation objects or a classification map back to the image.

For headless script execution, the *GroovyExecutor* class takes the URL of a Groovy-Script as argument and executes the Groovy code. This allows Orbit to be run on a server or HPC cluster and to submit a script which is located in e.g. a source control system. This feature is supplementary to the map-reduce principle; in fact, a script itself can start map-reduce processes and thus run analysis tasks at scale.

### Quantification methods

Orbit supports a variety of quantification methods such as pixel classification, object segmentation and object classification. These methods can be combined and applied on several resolutions of the image. For instance, the pixel classification method can be applied on a very low resolution of an image to define a valid ROI and then another method can be applied on high resolution within that ROI. Non-specialist users can train such machine learning models with only knowledge of pathology or biology, and no knowledge of machine learning, simply by using the graphical use interface (GUI).

#### Pixel classification

One of the basic principles in Orbit is the classification of pixels. Therefore, the user defines a set of classes, e.g. ‘background’, ‘staining 1’, ‘staining 2’ and assigns a colour to each class. One important parameter to set is the so-called structure size. This parameter defines the size of the surrounding context taken into account for each pixel. For each class, the user draws several shapes using a polygon, rectangle or circle tool. The union of all pixels inside these training-shapes define the training set. Several images can be used to define the training set with the goal of covering the entire variability of the tissue structures. For each pixel inside the training set, a feature extraction is performed, which takes into account not just the pixel itself, but a pre-defined area around each pixel delimited by the structure size. Based on this data the following features are computed: min, max, mean, standard deviation, edge-factor, and the middle pixel intensity per channel. For example, the edge-factor for pixel *p* characterizes the edginess and is defined as

$${\mathrm{edge}}_{p}=\sqrt{\frac{1}{|W|-1}\sum_{p^{'}\in W}{\left(p^{'}-p\right)}^{2}}$$ (1)

*W* defines the set of pixels inside the structure-size window around pixel *p*.

These features are used as input for a linear SVM which is used to train a statistical model for pixel classification. This circumvents the need for the user to specify any thresholds, as the tissue class discrimination is done simply by drawing shapes on representative regions. The structure-size here is very important: smaller values discriminate more fine-grained structures, higher values take more surrounding context into account. Orbit always makes use of all computed features and leaves to the SVM to weight the features appropriately. A maximum of 40,000 pixels is randomly picked from all training regions in order to perform a fast training. The training-set accuracy is computed internally (and shown in the log) on the training-set, without cross-validation. This explains the accuracy at which the model can discriminate the training classes and a warning popup appears if the accuracy is below 0.85, which indicates when the training regions should be placed differently. Usually a very small set of training regions per class is sufficient. However, Orbit allows training to be performed across several images in case there is a variation from image to image (e.g. variations in intensity). Comparable approaches like fastER [28] work in a similar manner and may be incorporated in future versions of Orbit.

#### Object segmentation

**Fig 3:**
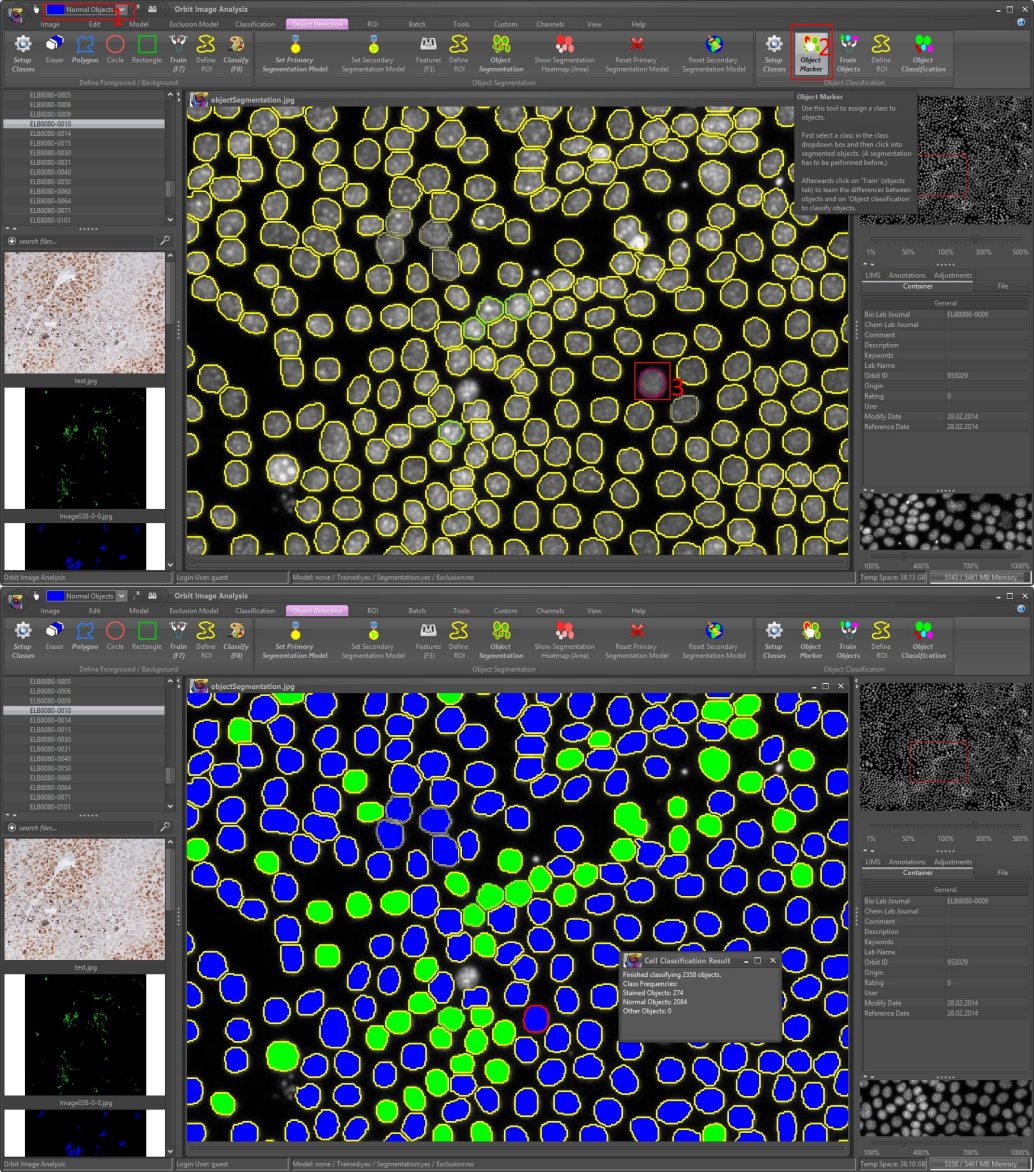
Object classification. Upper image: After an object segmentation the user can define object classes (1), use the object marker tool (2) and mark segmented objects (3). Orbit computes features for each object and uses a SVM to classify the objects. Lower image: Results of the object classification.

An object segmentation task groups the pixels that define an object, and requires a definition of foreground and background pixels, which can be determined from a pixel classification model that is run as a preceding step. Object segmentation in the biology domain is mainly used to segment cells, as visualized in Fig 3. Here the main challenge, especially in tissues, is that cells often overlap. Orbit provides a standard watershed algorithm [29] in combination with erosion and dilation steps which solves most of the problems. For challenging problems like dense cell clusters, a Mumford-Shah [30] functional-based segmentation algorithm can be used.

Two-level segmentation performs a second segmentation within segmented objects. For this, a second classification model is built and applied within the detected objects of the primary segmentation model. This is very useful for e.g. mRNA detection which is visible as small spots inside cells.

By default, Orbit simply outputs the number of segmented objects; however, in the segmentation settings the *Features* option can be activated. This enables the feature computation per segmented object which can work with several other parameters like shape descriptors, texture descriptors, location information, and outputs this information per segmented object.

#### Object classification

A machine-learning based object classification can be applied after the object segmentation. The idea is that the user can mark segmented objects and assign them to specific classes—without thinking about how to describe the differences between the object classes. To achieve this, Orbit provides an *object marker* tool which can be used after a segmentation step. For each class, the user marks several objects which defines the training set. Orbit internally computes the object features per segmented shape which will be used to train a SVM. Based on this statistical model, all segmented objects are labelled. The object classification can be seen as an additional step to the object segmentation. Sometimes this is very useful for discriminating objects, sometimes it is more appropriate to analyse the output of the object features with external data analysis tools.

#### Region of interest

The region of interest (ROI) is the area in which other algorithms are applied, e.g. an object segmentation only segments objects within the valid ROI and skips the other parts of a WSI. Usually the ROI defines the tissue and excludes the background, but it can also exclude unwanted parts like holes or wrinkles.

#### Annotations

Manual annotations can be drawn in the annotation panel using annotation tools like a pencil or a magnetic lasso. By default, an annotation can have a name and is just an informative annotation. However, annotations can also be edited and assigned a special type. Annotations of the type *ROI* define the region of interest (e.g. area outside the ROIs is excluded). If multiple ROIs are created, the final ROI for the image is the union of all ROI annotations. *Exclusion* annotations define excluded areas (e.g. inside a ROI annotation), unless another annotation of type *inclusion* exists. Inclusion annotations have the highest priority and overrule exclusion annotations. All created annotations are saved in the database and are automatically uploaded whenever an image is accessed.

#### Exclusion models

Technically an exclusion model is a classification model on a low resolution (approx. one megapixel) with a high structure-size. For each class, the user can define whether it is an inclusion or exclusion class. The training is done the same way as for a standard classification model, but a special exclusion model training automatically uses a low resolution of the image and later maps it to the full resolution. Manual annotations (e.g. exclusion annotations) and exclusion models can be combined, so some parts of a WSI can be explicitly excluded, even if the exclusion model would include it.

#### Masks

The masking functionality in Orbit is a generic approach for defining areas which are included and those which are excluded from analysis. In contrast to the exclusion model and manual annotations, masks do not affect the measured size of a ROI.

A classification mask is a standard pixel classification model with additional flags for each class defining whether the class should be included or excluded. In contrast to an exclusion model, the main model usually is applied on a high image resolution. The classification of the pixels is performed on-the-fly tile-by-tile when the main analysis on the particular tile is performed. One characteristic here is that the mask classification and the main analysis can be applied on different channels. This allows a channel co-localization quantification as is often used in fluorescence imaging.

A segmentation mask is a segmentation model in which all pixels inside segmented objects are declared as active pixels used by further analysis. This allows a pixel classification inside segmented objects, or a nested segmentation.

#### Tools

Orbit provides many other tools out-of-the-box to cope with the most common image analysis problems. These include (among others) a tool to run Cell Profiler pipelines within the ROI defined by Orbit and read back the cell segmentations, a nerve fibre detection tool, a tissue microarray (TMA) spot detection tool and a rare event detection tool. The latter is a very flexible tool which performs an object segmentation and presents all detected objects to the user who then can manually assign each object to a class. A report outputs the frequencies of the assigned classes.

In addition to the built-in tools, image analysis specialists can create custom tools which are instantiated via Java reflection and listed as ribbons in the *custom* tab. The developer has access to all open images, annotations and models in order to perform an analysis task.

## Results

Orbit has been applied for solving many real-world imaging analyses problems in the drug discovery and medical domains [31,32,33,34]. Here, we briefly describe the results of two of these (idiopathic pulmonary fibrosis quantification, intraepidermal nerve fibre detection) and describe a third (glomeruli detection), which is based on deep learning, in greater detail.

### Application 1: Idiopathic pulmonary fibrosis quantification

**Fig 4:**
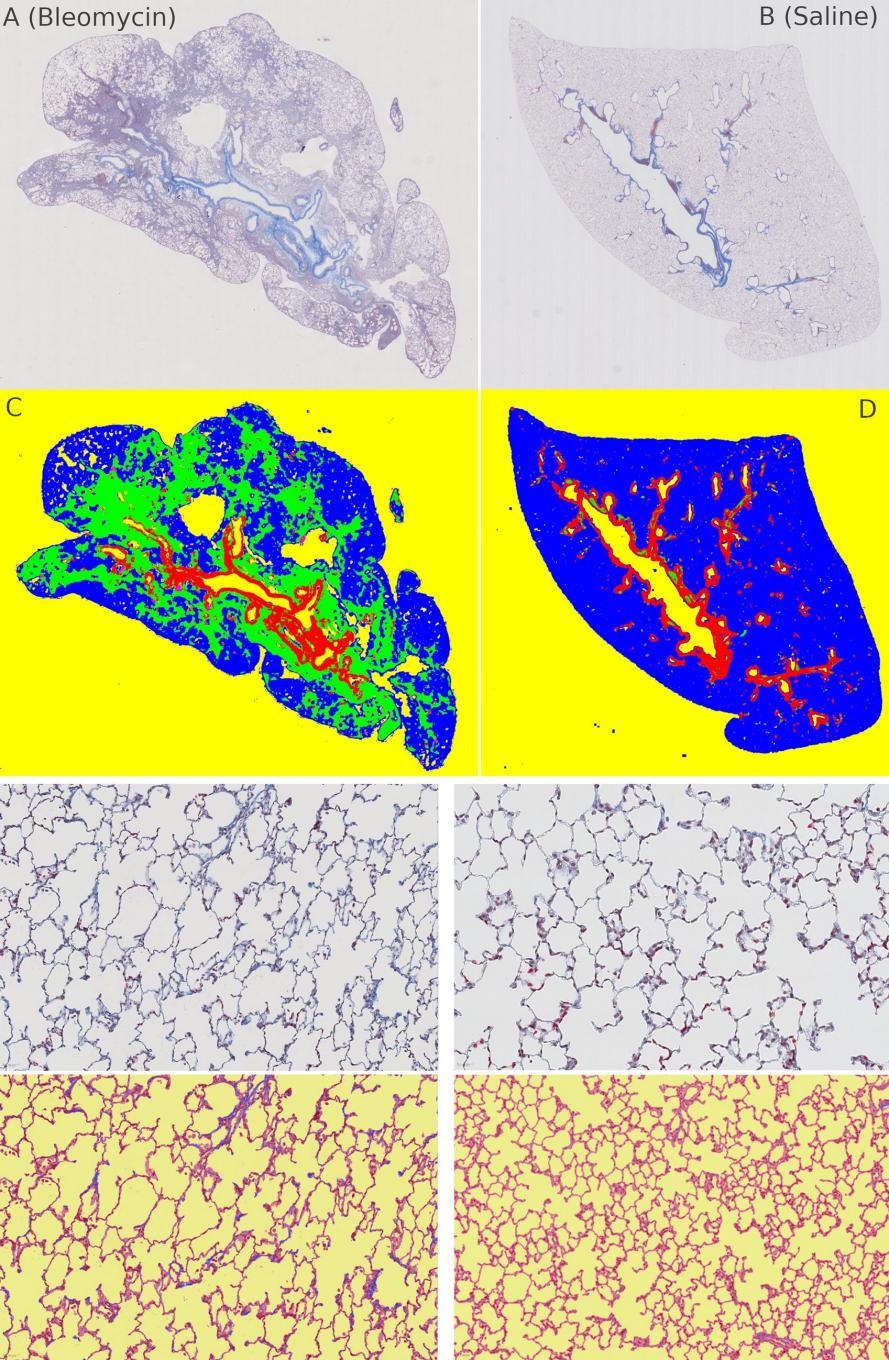
Idiopathic Pulmonary Fibrosis Quantification (IPF). IPF quantification: Treated/unhealthy (left) and control/healthy (right). (A,C) and (B,D) shows how the exclusion model works. (E,G) and (F,H) shows the fine-grained collagen quantification.

Idiopathic Pulmonary Fibrosis (IPF) is a life-threatening disease where collagen deposits in the lungs lead to a shortcoming in the blood-oxygen exchange. Lung tissue sections are stained with Masson’s Trichrome stain to mark the collagen on the slide (Fig 4). Evidence for IPF are collagen deposits along alveoli walls and the presence of dense fibrotic areas (sometimes called fibrotic foci).

The quantification of fibrotic collagen deposits along alveoli walls is therefore a key step in the diagnosis of IPF. However, there are two major hurdles to overcome. First, it is important to differentiate between the structural collagen which is normally present along the vessel borders versus pathological collagen deposits. Masson’s Trichrome stain, however, does not distinguish between normal structural collagen and the fibrotic collagen deposits in alveoli walls. Both have the same staining colour (blue). Second, there is a high staining variability between images from different batches of slides. This inherent heterogeneity is due to the use of different staining machines as well as inter-user variability. The current gold standard is a manually performed analysis of Masson’s Trichrome-stained paraffin sections using a scoring system defined by Ashcroft et al. [35]. Today, with the combination of WSI scanners and the availability of huge computation power, Orbit can be used to automatically, objectively and reproducibly quantify IPF images [36,32].

For this type of analysis, the Orbit workflow employs a combination of an exclusion model and a classification model. The exclusion model consists of four classes: two exclusion classes (background and vessel border), and two inclusion classes (normal tissue and fibrotic area). Normally, an exclusion model is only used to define a ROI, however here the ratio of the fibrotic area is used as a result.

A classification model which discriminates between background, tissue and collagen is applied inside the valid ROI defined by the exclusion model. The collagen along the alveoli walls and the collagen around the vessels can be distinguished because the exclusion model is applied on low magnification (~5x), and the classification model on high magnification (20x or 40x) of the image. The ratio of alveoli wall collagen is used as a second result.

Both output parameters, fibrotic area and collagen, can be used individually or as an input for a linear SVM to predict the real IPF scores which have been manually pre-defined by pathologists. Stritt et al. reported in [36] that using this method a correlation factor of 0.81 can be achieved. In [31] the authors show that by using a larger training set, a model can be created which yields robust results for a large series of images gathered over a period of five years with high staining variability.

### Application 2: Intraepidermal nerve fibre density quantification

Intraepidermal nerve fibre density (IENFD) is a biomarker for peripheral neuropathies, defined by the number of nerve fibres crossing the basement membrane between the dermis and epidermis per millimetre [37]. The gold standard so far is the manual assessment of IENFD under a microscope according to a set of established guidelines [38]. The aim of Seger et al. [32] was to standardize the quantification of IENFD in human skin biopsies and to therefore decrease the inter-rater variability.

Based on many skin biopsy images, an Orbit model was created to recognize the nerve fibres. A special segmentation variation based on a high and low threshold edge detection algorithm, similar to the Hysteresis plugin of ImageJ, is used to segment and connect nerve fibres. Low threshold parts connected to high threshold parts are connected to high threshold objects, the other stand-alone low threshold parts are discarded. Technically this segmentation step is a map step. The reduce step connects partial nerve segments which cross tile borders.

**Fig 5:**
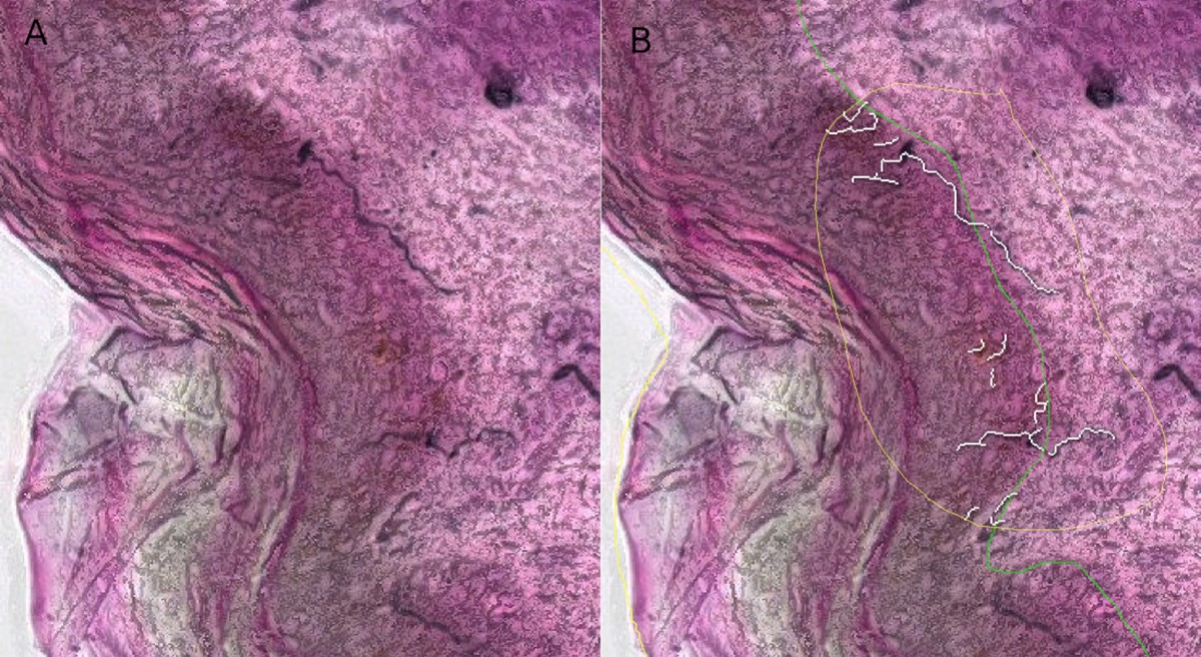
Intraepidermal nerve fibre detection. Nerve fibre image (A) and annotated automatically detected nerves (B) on a bright-field image. Only nerve fibres close to or crossing the manually annotated junction line are detected.

The model including the threshold parameters is very robust [32] and can be applied to fluorescence and bright-field stained skin biopsies (Fig 5). It is freely available and included in the nerve fibre detection module in Orbit.

After automated nerve fibre detection, all segments crossing the dermal–epidermal junction are randomized and presented to the user as a list. By clicking on an element the corresponding nerve fibre segment is centred on the screen and the user has the option to either skip it in case of false detection, or score the number of junction crossings by pressing a number key.

This method leads to a very robust and unbiased (randomized sections, each section presented only once) quantification which decreases the inter-rater variability.

### Application 3: Glomeruli detection

**Fig 6:**
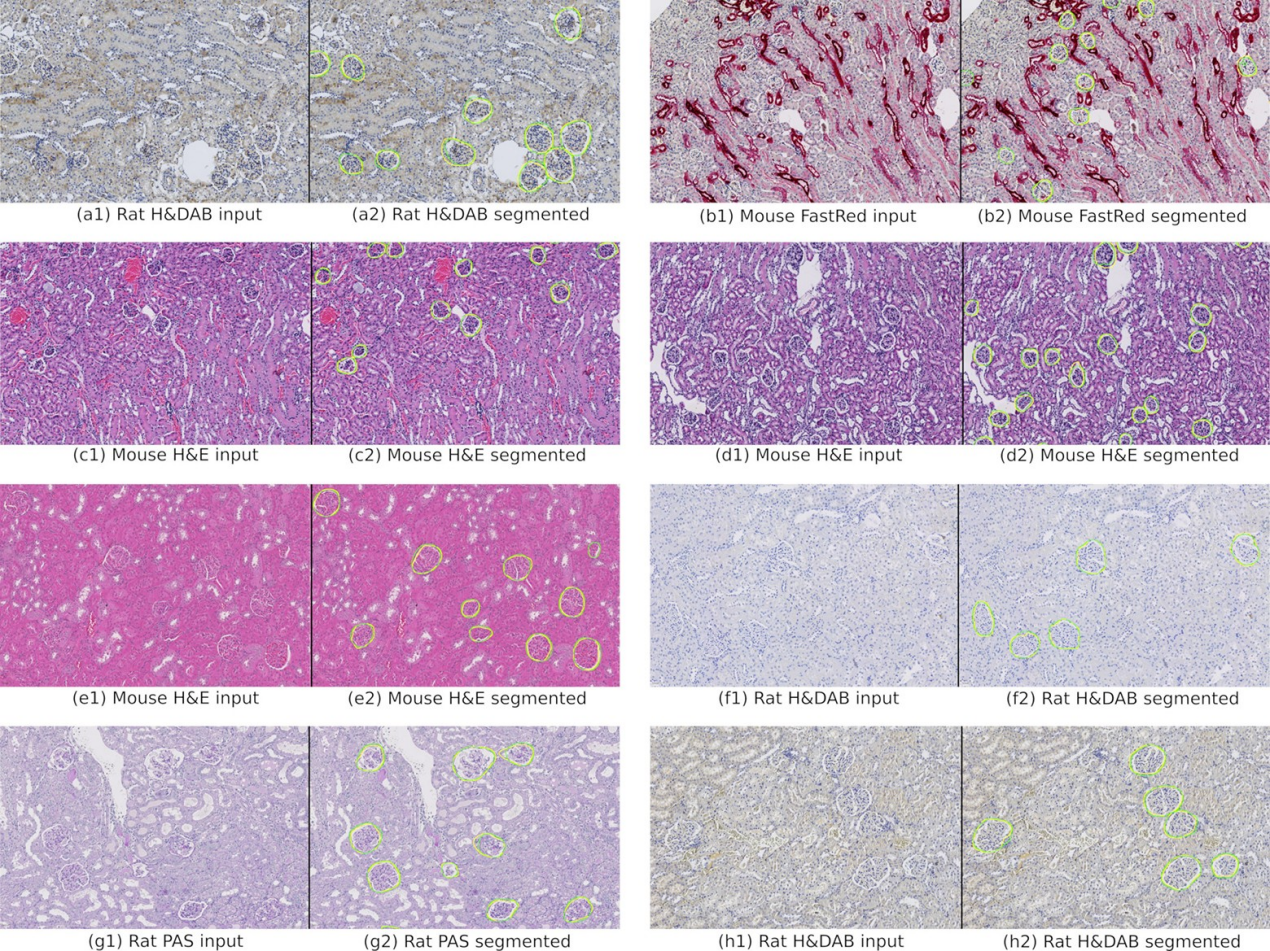
Glomeruli detection on kidney slides using deep learning. Manually annotated glomeruli outlines (ground truth) in yellow, detected glomeruli outlines in green.

Standard object detection methods allow the detection of homogeneous objects like cells, but fail on complex heterogeneous objects where the larger context plays an important role. A good example for this is glomeruli detection, which is important for quantifying various kidney diseases. Glomeruli in kidney tissue are heterogeneous objects with a high variability (Fig 6). For detecting these objects, we apply a deep learning segmentation approach based on an encoder-decoder convolutional neural network (CNN) structure. This approach is generic and can also be used to detect objects other than glomeruli [14,22]. We provide a pre-trained model for glomeruli detection which is ready to use for segmentation. It can be downloaded from the Orbit model zoo: https://www.orbit.bio/deep-learning-models/. For detecting other objects the user can build a new training set based on manually annotated objects, which can then be used to train a new segmentation model. Orbit provides scripts (see https://www.orbit.bio/deep-learning-object-segmentation/) to train a new CNN and user-friendly methods integrated within the user interface for those who wish to apply a pre-trained CNN.

#### Training

To build a new deep learning model from scratch, the first step is to annotate a set of representative objects over several images using the annotation tools. For the glomeruli example we annotated 92 slide images resulting in around 21,000 annotations. This huge amount of annotations was necessary because the aim was to create a model which is able to cope with two species (mouse and rat) and several different staining protocols: H&DAB, FastRed, PAS, and three variations of H&E. The ultimate goal was to detect glomeruli on any WSI. In addition to the object annotations, we drew a ROI annotation to define the region which we considered for the annotations. This implies that outside the ROI there might be further objects which are not taken into account.

**Fig 7:**
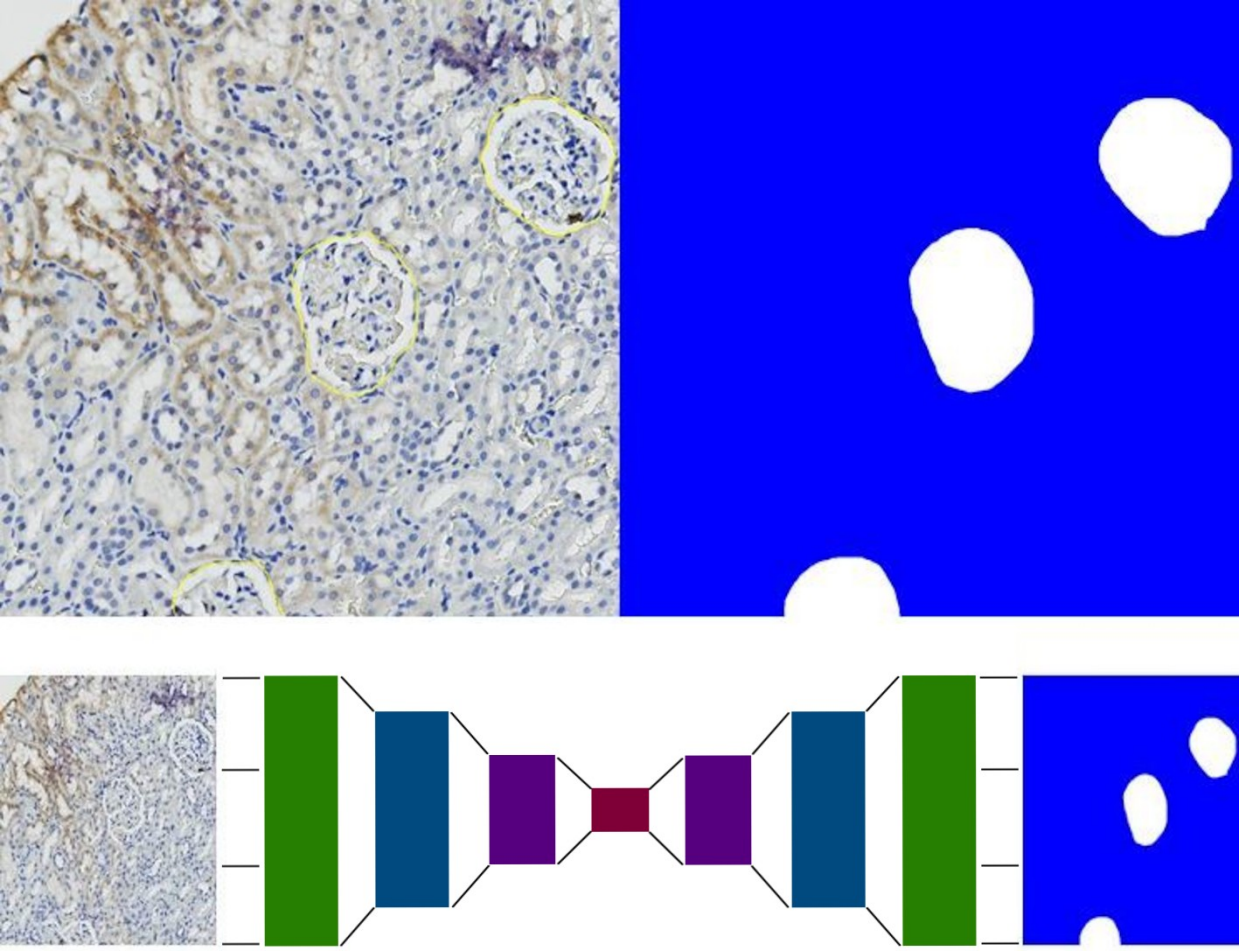
Deep-learning object detection. Top: Annotated objects on the left side, generated segmentation map which is used as training input on the right side. Bottom: The ResNet-101 based encoder-decoder network takes a tile image as input and outputs the tile mask.

Orbit generates 512x512 pixel-sized tile masks which encode objects and background out of the manually drawn annotations (Fig 7). These masks act as the training set for the actual training of the deep learning model. We provide a Python script for training which has to be executed outside of Orbit, ideally on a machine with a GPU (https://www.orbit.bio/deep-learning-object-segmentation/). The script uses TensorFlow as a deep learning framework and runs out-of-the-box, where only the file path of the tile masks has to be defined. As a base CNN we use a ResNet-101 network structure [39] pre-trained on the MSCOCO dataset [23]. The input tensor is a tile image and the output (target value) is its corresponding tile mask. The encoder-decoder network structure is depicted in Fig 7.

#### Segmentation

Once the model is created, or a pre-trained model downloaded, objects on images can be segmented. As a first step the CNN model was used to predict the tile masks. We observed that the model detected objects very well if they were located at the centre of the tile, but often only partially when they were located close to the tile border. Fortunately, the false-positive pixel-rate in the tile mask was close to zero, indicating that the model correctly detected a pixel inside an object—but just missed some. This highlighted the need for a segmentation refinement step: arbitrary translations were applied to the tile, for each variant the tile mask was predicted, and the refined result mask was the union of all variant masks with respect to their inverse translation. For the glomeruli detection, four translations were applied: half the tile size up, down, left and right. Based on the tile mask, the object segmentation was performed using a pre-defined segmentation model. Another refinement was applied to cope with tile-crossing objects: each segmented object was centred, a virtual tile around the centre of the object was extracted and used as input for another segmentation step. The idea was that partially segmented objects were to be detected completely if they were located at the centre of the tile. All segmented objects were stored in an object list. A post-processing step eliminated duplicate objects from that list by only keeping the ones which cover others.

#### Evaluation

The complete dataset consisted of 90 whole slide kidney images from rat and mouse samples and several types of stains (https://doi.org/10.5061/dryad.fqz612jpc). Fig 6 shows the huge variability between the stainings. For this evaluation, the data set was divided into 58 training and 32 test images. Each slide image contained around 300 glomeruli objects. For each group (species x staining) the test samples were independent but randomly selected to ensure a good stratification. The training set was virtually enhanced with 90- and 180-degree rotation images in order to achieve rotation invariance. The complete training set including the rotations contained 87213 images and masks, each 512x512 pixels. The training of the network was performed on a high-end gaming GPU and lasted 4 days for 410,000 training iterations with a batch size of 2.

The evaluation of the test set was based on the Dice index [40] which scores the overlap between pixels in the range of [0,1] where 1 means a complete overlap. For each ground truth object, the closest segmented object was taken into account and vice versa. The sum of the two overlap ratios was divided by two. We used the dice object index as defined in [41] to score a complete image by using the Dice index for all objects, weighted by its object size.

Table 1 shows the Dice object index results per group and the total. The scores show a good segmentation performance over a set of 32 whole slide images, each containing around 300 glomeruli. This numeric evaluation has been confirmed by a subjective visual evaluation; an example is shown in Fig 6.

**Table 1:** Evaluation results of the glomeruli segmentation for each group and total. Samples of each staining type are illustrated in Fig 6.

Type	Mean Dice Obj. Index ± SD	#Images
H&DAB Rat 1	0.8299±0.0194	4
H&DAB Rat 2	0.8975±0.0100	4
H&DAB Rat 3	0.8837±0.0245	4
FastRed Mouse	0.8225±0.0319	4
PAS Rat	0.9092±0.0129	4
H&E Mouse 1	0.9030±0.0123	4
H&E Mouse 2	0.8807±0.0203	4
H&E Mouse 3	0.8768±0.0135	4
Total	0.8754±0.0181	32

Note that the ground truth was defined by manual annotations which might not be fully accurate or may miss some glomeruli. Indeed, a visual inspection did show that the automated detection found some new glomeruli which had not been annotated. Interestingly, the models performed very well despite a high staining variability, e.g. H&E images greatly differ from FastRed and H&DAB images, but the model was able to detect glomeruli regardless of the staining protocol used.

## Discussion

We describe the successful application of Orbit across three completely different image analysis scenarios. The quantification of lung fibrosis represents a structure discrimination task that necessitates combining several magnification levels of a WSI. The second task (intraepidermal nerve fibre density quantification) demonstrated the challenge of segmentation across image tiles and combining it with semi-automated analysis methods: the computer presents segments to the user, and the user manually applies a grade. This is only possible because of the functional harmony between the image analysis engine and the user interface.

The third application demonstrates the integration of a complex deep-learning model to identify glomeruli objects in kidney. This showcases two powerful capabilities of Orbit: the integration of external tools, and the combining of the partial (tile-based) analysis results. Here we used TensorFlow to train the deep learning model and also as an inference engine. The Orbit tile-processing engine then combined the partial results per tile. Only with that approach was it possible to detect glomeruli across tile borders. Lutnick et al. [42] published a similar workflow for glomeruli detection based on the combination of the tool ImageScope from Aperio and a DeepLab V2 network. In contrast to our approach, the authors provide an interactive workflow which allows experts to correct annotations during multiple iterations. Since the ImageScope tool is closed source and not applicable to all WSI formats, it would make sense to integrate the interactive workflow of Lutnick et al. into Orbit in the future.

In conclusion, Orbit harnesses the power of context-based structure classification alongside generic tile-processing, whilst enabling the integration of different algorithms. Existing algorithms designed for use with smaller images can be applied towards very large images. The scale-out features (e.g. using Apache Spark) allows the scaling-up and processing of hundreds of WSI in parallel. The generality of its tile-processing engine is complemented by sophisticated machine-learning algorithms. In addition, the user-friendly scripting capability simplifies the workflow for multiple end-users, from specialist script writers to biologists with no knowledge of programming. Altogether, these key characteristics of Orbit render it a powerful tool to meet the rapidly-growing needs of digital pathology.

## Availability and future directions

The main website for Orbit is https://www.orbit.bio where the program itself, models, tutorials, and documentation can be found.

### Binaries

At https://www.orbit.bio/download the pre-compiled binaries can be downloaded for all major plattforms: Linux, Mac and Windows, licensed under the GPLv3.

### Source-code

The source-code is available at https://github.com/mstritt/.

### Models

Standard pre-trained Orbit models can be found at https://www.orbit.bio/orbit-models/, deep-learning models at https://www.orbit.bio/deep-learning-models/.

### Image data

The glomeruli image dataset and annotations are available at https://doi.org/10.5061/dryad.fqz612jpc.

### Documentation & support

The documentation area at https://www.orbit.bio provides a detailed explanation of the workflows and procedures. Some tutorials can be found at https://www.orbit.bio/help/ and the handbook is available at https://www.orbit.bio/orbithandbook/. For further support, requests and discussions, please refer to the forum https://forum.image.sc/tags/orbit.

### Future directions

Future work will improve Orbit in two ways: basic functional enhancements for a better user experience, and more powerful quantification and segmentation methods such as the integration of the most recent deep learning network architectures. Relevant feedback from the highly active user community will be used to incorporate practical requests and develop solutions in an agile development process.
